# Emergency Contraceptives: Knowledge and Practice towards Its Use among Ethiopian Female College Graduating Students

**DOI:** 10.1155/2019/9397876

**Published:** 2019-01-01

**Authors:** Kirubel Minsamo Mishore, Abebaw Demissie Woldemariam, Solomon Assefa Huluka

**Affiliations:** ^1^School of Pharmacy, Haramaya University, Harar, Ethiopia; ^2^Department of Anesthesia, Harar Health Sciences College, Harar, Ethiopia; ^3^Department of Pharmacology and Clinical Pharmacy, School of Pharmacy, Addis Ababa University, Addis Ababa, Ethiopia

## Abstract

**Introduction:**

Ethiopia has a high incidence of unwanted pregnancies and incomplete and unsafe abortions, particularly among adolescents. This can be avoided by using different contraceptive methods including emergency contraceptives (EC). This study aimed to assess the knowledge and practice of EC among female college graduating students in Harar, Eastern Ethiopia.

**Methods:**

Institution based cross-sectional study was conducted on 214 female students selected from two randomly selected colleges. Data was collected using a self-administered questionnaire and analyzed using SPSS for Windows version 20.1. Level of significance was taken at P <0.05.

**Results:**

The mean (±SD) age of the participants was 21.06 (±2.14) years. Of the 200 (93.5%) study participants who had ever heard of ECs, 140 (70.0%) had good knowledge. Among the 214 graduating female students, 66 (33.0%) had ever used EC. Being above the age of 20 years old, father's and mother's literacy were found to be determinants of knowledge of EC. Moreover, knowledge was the only determinant factor of practice of EC.

**Conclusion:**

Most of the respondents had relatively good knowledge of EC. The study revealed that female students of older age and higher educational status of parents had higher knowledge and practice of EC.

## 1. Introduction

Unintended pregnancy and unsafe abortion can be prevented by access to contraceptive methods including emergency contraceptives (EC). EC is a method used to avoid pregnancy after unprotected sexual intercourse. It is used before the potential time of implantation, unlike the regular contraceptive methods that are administered before the act [1]. EC can reduce the risk of unintended pregnancy by 75% to 99% if taken within 72 hours of sexual intercourse [2, 3]. ECs are cost-effective, medically safe, and highly effective to be used for the prevention of unplanned pregnancy and subsequently avoid unsafe abortion and other consequences [4].

An estimated 80 million unintended pregnancies occurred in 2012 in the developing world, resulting in 40 million abortions and 10 million miscarriages [5]. About one-third of unintended pregnancies each year result from the incorrect use or failure of contraceptives [6]. World Health Organization (WHO) estimates that every year, nearly 5.5 million African women have unsafe abortions. Moreover, 59% of all unsafe abortions in Africa are among young women aged 15-24 years [7].

Unsafe abortion due to an unplanned pregnancy is one of the main causes of maternal morbidity and mortality in Ethiopian women [8, 9]. Several studies in the country have revealed that women who tend to undergo induced abortions are below the age of 30 and above the secondary educational level [10–12]. Young people today start sex before marriage. Thus, they face a greater risk of unintended pregnancy [13].

Studies conducted in Ethiopia [1, 14–16] indicated that awareness of EC is less than 50% and utilization is below 10%. These limited studies conducted on the issue of EC in the country were mostly focused on university students, who are believed to have better overall knowledge than college students [17]. Studies conducted in college students were not specific to their year of study, which was found to be determinant of knowledge of EC among female college students [18]. Thus, the aim of the present study was to assess determinants of knowledge and practice of ECs among female college graduating students in Harar town, eastern Ethiopia.

## 2. Methods 

### 2.1. Study Setting

The study was conducted in Harar town, eastern Ethiopia, which is located 525 kilometers east of Addis Ababa. There are eight colleges (private and governmental) in the city. Harar Health Science College (HHSC), Harar Poly Technique College, and Harar Teachers Training College are governmental colleges. Rift Valley University Harar Campus (RVUHC), AferenQallo College, Horn International College, Dakmas College, and Lucy University College are privately owned colleges. Two of the colleges, namely, HHSC and RVUHC, were selected by simple random sampling methods from the list.

### 2.2. Study Design and Data Collection

**Figure 1:**
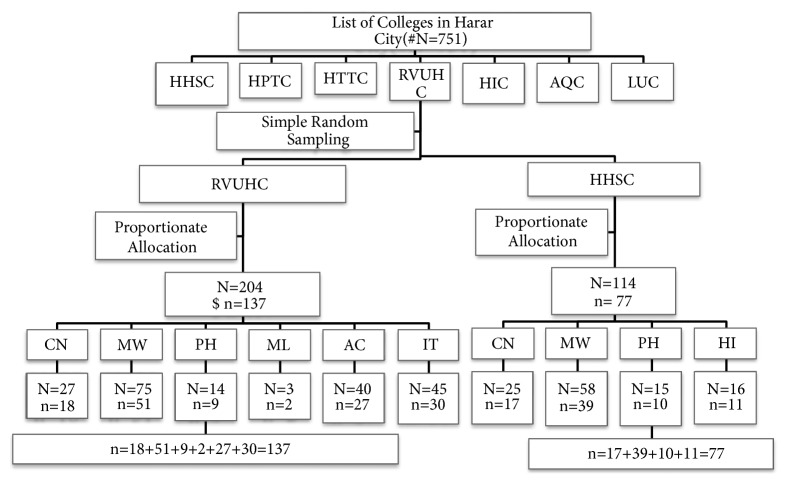
Schematic presentation of sampling procedure. # Total number of graduating class female students in each college and departments. $ Sample size proportionally allocated to each college and departments. Comprehensive Nurse (CN); Midwifery (MW); Pharmacy (PH); Medical Laboratory (ML); Accounting (AC); Information Technology (IT); Health Informatics (HI).

An institution based cross-sectional study was conducted in June 2016 among graduating female college students from selected colleges in Harar town. Sample size was determined using the simple proportion formula for cross-sectional study and taking the proportion of 21.9% for good knowledge of EC [18]. The multistage sampling techniques were used to approach the participants. Since the source population was less than 10,000, finite population correction was made, which brought the sample size to 214. Then, the sample size to each selected college and respective departments was then allocated proportionally using total number of female graduating students. Finally, the study participants were selected randomly (Figure 1). The study was conducted by means of a self-administered questionnaire survey. The questionnaire included questions on demographic variables, sexual practice, and knowledge and practice of EC.

### 2.3. Data Analysis

Following data collection, a unique code was assigned to each questionnaire. Data was then entered, cleaned, and imported to SPSS version 20.1 for analysis. Both bivariate and multivariate analysis techniques were applied to identify the factors associated with knowledge towards EC. The variables that were significant in the bivariate analysis were reexamined using stepwise binary logistic regression, to identify the significant predictors after controlling for other variables.* P* values less than 0.05 were considered as statistically significant.

The respondent's knowledge scores were aggregated and ranged 0–9. Based on the cumulative score, respondents who scored 5 (mean score) and above were considered to have “good knowledge”, while those who scored below the mean score were considered to have “poor knowledge”. Practice was determined based on every use of EC after exposure to unprotected sexual intercourse to prevent unintended pregnancy.

### 2.4. Ethical Consideration

The study was carried out after obtaining ethical clearance from Institutional Ethical Clearance Board of Harar Health Science College and permission from the dean offices of selected colleges. All participants were informed about the objectives and their right to leave the study at any time they wished. A verbal consent was obtained from all participants. Confidentiality of the participants was maintained at all times. To further maintain anonymity, no forms of identifiers were in the questionnaires, as code numbers were used and only data collector and supervisor were involved in the data collection and supervision process.

## 3. Results

### 3.1. Sociodemographic Characteristics

A total of 214 female students of the graduating class participated in the study. The mean (±SD) age of the participants was 21.06 years (± 2.14). Ninety (42.1%) of them were midwifery students followed by nursing students (16.4%). As it is shown in Table 1, most of the respondents 164 (76.6%) were urban residents. Nearly three fourths (74.3%) of the respondents were unmarried. The majority (132; 61.7%) of the participants were living with their parents (Table 1).

### 3.2. Knowledge of Emergency Contraceptives

**Figure 2:**
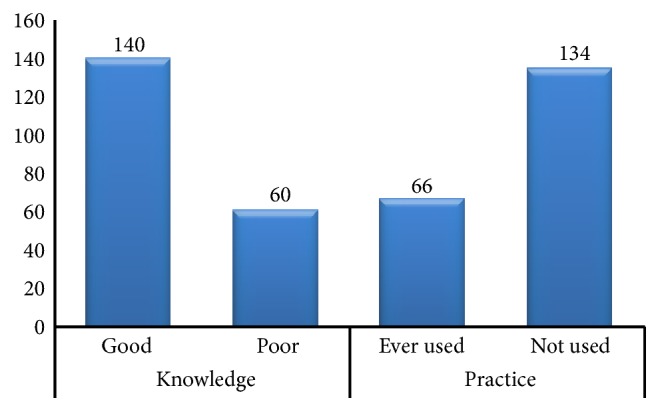
Frequency distribution of knowledge and practice of emergency contraceptive among female college graduating students of Harar, eastern Ethiopia, 2016(N=200).

Majority of the participants (93.5%) reported that they had heard about EC. Only those respondents who had heard of EC (200) were further analyzed for having knowledge and practices. The overall summary index for knowledge disclosed that 140 (70.0%) of the study participants had good knowledge about EC (Figure 2). The main sources of information about EC were college (40.5%) followed by healthcare workers (29%) and mass media (24.5%). Most of the respondents 130 (65.0%) could identify the correct timing for administration of emergency contraceptive pills after unprotected sex. However, only 54 (27.0%) could tell the correct timing of insertion of the intrauterine contraceptive device (IUCD) (Table 2).

### 3.3. Practice of Emergency Contraception and Sexual Activity

Among female graduating college students who had ever heard of EC, almost one-third (33.0%) of them had ever used EC Pills (Figure 2). However, none of the women had ever used IUCD. Pills missed, failed withdrawal, timing miscalculation, and condom slippage were the commonly stated reasons for using EC each accounted for 15 (22.7%), 9 (14.0%), 9 (13.6%), and 8 (12.1%), respectively. Seventy-one (55.9%) of participants started sex below age of 20 years. Among the sexually active students, 30 (23.6%) had an experience of pregnancy. Unintended pregnancy were observed in 20 (15.7%) of participants (Table 3).

### 3.4. Determinants on Knowledge and Practice of EC

Sociodemographic factors, age and educational status of the respondents' parents, showed a significant association with good knowledge of EC in multivariate logistic regression analysis. Females who were older (>20 years) were 6.09 times more likely to have good knowledge of EC than their younger counterparts (AOR: 6.09, 95% CI: 2.89-12.86, P≤0.001). On the other hand, respondents with a literate father were 3.5 times more likely to have good knowledge of EC compared to those with a nonliterate father (AOR: 3.50, 95% CI: 1.12-10.94, P=0.031). Similarly, respondents whose mother was literate were 2.84 times more likely to be knowledgeable (AOR: 2.84, 95% CI: 1.02-7.88, P=0.046) compared to their counterparts (Table 4).

Among variables, which showed association with bivariate logistic regression analysis, only having good knowledge of EC showed a significant association with student's ever utilization or practice of EC in multivariate analysis. Accordingly, students who had good knowledge of EC were 2.53 times more likely to practice EC than those who had poor knowledge of EC (AOR: 2.53, 95%CI: 1.29, 4.92) (Table 5).

## 4. Discussion

Unlike other contraceptive medicines, EC is not given regularly. It is given in cases of unprotected sexual relations, which carry the risk of undesired pregnancy [16]. Awareness of the accessibility and being taken within the defined time period are necessary for appropriate and effective use of EC [19, 20].

Majority (94%) of the study participants included in the study were aged 24 or below, which is considered a sexually active age group [21]. This finding is also in line with a similar study done in Nigeria [22]. The overall prevalence of awareness among the study participants was 93.5%. This finding is higher as compared to studies done in different parts of Ethiopia: Haramaya (47.6%)[14], Adama [15] (46.8%), Jimma (41.9%) [16], Ambo (80.7%) [17], Arba-Minch (42.5%) [18], Hosanna (45%) [23], and Debre-Markos (71.1%)[24]. However, this finding is comparable to the result of studies in Mekele (90.7%). Similar levels of awareness prevalence were also recorded in India (92.7%) [25, 26]. In the present study, the major source of information about EC was college, unlike a study done in Addis Ababa [27] whereby participants relied on friends and family for knowledge of EC.

Nearly three quarters (70.0%) of the respondents had good knowledge of EC in this study, which is superior to the result obtained in the studies conducted among Ethiopian colleges and university female students in Haramaya (25.7 %)[14], Arba-Minch (21.9%) [18], and Debre-Markos (62.5%)[24], Mizantepi [28] and elsewhere in India [26] and Nepal [29]. This difference might be attributed to a better free discussion on sex and sexuality among female students in Harar. However, the finding is unexpectedly lower than a study conducted among female preparatory students in Mekelle where 75.7% of the students had good knowledge of EC[25]. Moreover, in this study 130 (65%) of the participants correctly identified the time limit of EC pill use, which is superior to a study done by Abate et al., 18.5% [30]. This could be due to the high health promotion and availability of EC in pharmacies found [31] in Harar.

The finding showed that unintended pregnancies were observed in 20 (15.7%) of the respondents, which is slightly lower than study conducted in northwest Ethiopia (16.5%) [31]. However, it is much lower than the result reported in the national figure EDHS 2011 (24%) [32], and GanjoWoreda (27%)[33]. In the present study, about a third (33.0%) of the respondents had ever used EC. This finding is higher than the study conducted in Dilla (20.9%) [34]. It is also higher than EC use reported in Nigeria and India [26, 35]. This may be explained by wide availability of EC in Ethiopia particularly in major cities [30] and longer college stay.

In this study, age of the student showed a statistically significant association with good knowledge of EC. In agreement with this study, multiple studies [14, 24, 25] have reported a significant association between older age and good knowledge of EC. However, other study done by Melkam et al. [19] reported a contrary finding. This study, furthermore, revealed that EC knowledge was significantly higher for respondents whose parents were literate compared to those with nonliterate parents. This finding is consistent with the conclusion made by different researchers [18, 24], in which literacy improves one's access to information and facilitates discussion of reproductive health issues in the household.

The present study also showed that female students who had adequate knowledge of EC were more likely to use EC than their counterparts, which was in agreement with studies from Ethiopia and other countries [18, 24, 26, 36].

## 5. Conclusion and Recommendation

In this study, respondents' knowledge of EC is found to be acceptable. This study revealed that age and parents' literacy showed statistically significant association with good knowledge of EC and in turn good knowledge of EC was the only determinant of using EC. To prevent unintended pregnancy among sexually active female college students, sexual education given at high school or counseling provided to college students is highly recommended.

## Figures and Tables

**Table 1:** Sociodemographic characteristics of female college graduating students in Harar town, eastern Ethiopia, 2016 (N=214).

Variable	Category	Frequency	Percent
**Age group (year)**	≤20	108	50.5
	21-24	93	43.5
	25^+^	13	6.1

**Residence**	Urban	164	76.6
	Rural	50	23.4

**Religion**	Christian	133	62.1
	Muslim	81	37.9

**Marital status**	Single	159	74.3
	Ever married	55	25.7

**Field of study**	Midwifery	90	42.1
	Comprehensive nurse	35	16.4
	Information technology	30	14
	Accounting	27	12.6
	Pharmacy	19	8.9
	Health informatics	11	5.1
	Medical laboratory	2	0.9

**Living with**	Parents	132	61.7
	Alone	34	15.9
	Husband	26	12.1
	Friends	22	10.3

**Father's Education**	Nonliterate	28	13.1
	Completed primary school	44	20.6
	Completed secondary school	59	27.6
	Completed tertiary school	83	38.8

**Mother's Education**	Nonliterate	34	15.9
	Completed primary school	51	23.8
	Completed secondary school	72	33.6
	Completed tertiary school	58	27.1

**Table 2:** Knowledge of emergency contraceptives among graduating female college students of Harar, eastern Ethiopia, 2016 (N=200).

Characteristics	Category	Frequency	Percent
**Ever heard of EC**	Yes	200	93.5
	No	14	6.5

**Source of information**	College	81	40.5
	Healthcare workers	58	29
	Mass media	49	24.5
	Others	12	6

**Method reported used as EC**	Pills	124	62.0
	IUCD	18	9.0
	Pills and IUCD	29	14.5
	Implants and Injectable	29	14.5

**Places where a woman can obtain EC**	Hospital /health center	109	54.5
	Pharmacy	59	29.5
	Don't know	10	5.0
	Others	22	11.0

**Indication of EC**	After unprotected sexual intercourse	114	57.0
	When unwanted pregnancy occurs	62	31.0
	As regular method of contraceptive	24	12.0

** Drug strength in EC pills compared to regular pills**	The same strength	92	43.0
	Higher dose in the same hormones	35	16.4
	Don't know	73	34.1

**Maximum time limit for taking ECPs **	Within 72 hours	130	65.0
	Within 48 hours	9	4.5
	Within 24 hours	10	5.0
	Within 12 hours	18	9.0
	Don't know	33	16.5

**Maximum time limit for having an IUCD after unprotected sex**	Within 5 days	54	27.0
	Within 72 hours	30	15.0
	Within 48 hours	12	6.0
	Don't know	104	52.0

**Effectiveness of EC methods in preventing pregnancy when used within the specified time limit**	<75%	20	10.0
	≥75% but < 100%	113	56.5
	Not sure	67	33.5

**How safe do you think EC are for most women?**	Safe	138	69.0
	Unsafe	28	14.0
	No response	34	17.0

**Table 3:** Practice of emergency contraception and sexual activity among female college graduating students, Harar, eastern Ethiopia, 2016 (N=200).

Variables	Category	Frequency	Percent
**Ever use of EC**	Yes	66	33
	No	134	67

**Frequency of use **	Once	30	45.5
	Twice	14	21.2
	Three times	7	10.6
	Not specified	15	22.7

**Recommended by**	Friends	20	30.3
	Partner	18	27.3
	Health professional	23	34.8
	Not specified	5	7.6

**Reasons for using**	Timing miscalculation	9	13.6
	Condom slippage/breakage	8	12.1
	Pills missed	15	22.7
	Forced sex	6	9.1
	Failed withdrawal	9	13.6
	Not specified	19	28.8

**History of sexual practice**	Yes	127	63.5
	No	73	36.5

**Age at first sex**	15-19	71	55.9
	≥20	47	37.0
	Not specified	9	7.1

**History of pregnancy**	Yes	30	23.6
	No	97	76.4

**Was the pregnancy planned?**	Yes	10	33.3
	No	20	66.7

**Table 4:** Univariate and multivariate analysis of factors associated with knowledge of EC among female college graduating students, Harar, eastern Ethiopia, 2016 (N=200).

Variable	Category	Knowledge		COR (95% CI)	*P* value*∗*	AOR (95% CI)	*P* value*∗*
		Good	Poor				
**Age**	≤20	52	441.00			1.00	
	>20	88	16	4.65(2.39-9.07)	0.001	6.09(2.89-12.86)	0.001

**Marital Status**	Unmarried	96	50	1.00		1.00	
	Ever married	44	10	2.29(1.06-4.94)	0.034	1.83(0.64-5.26)	0.261

**Field of Study**	Health	113	40	2.1(1.06-4.12)	0.034	1.87(0.82-4.3)	0.14
	Not Health	27	20	1.00		1.00	

**Fathers' Education**	Nonliterate	10	16	1.00		1.00	
	Literate	130	44	4.73(1.99-11.18)	0.001	3.50(1.12-10.94)	0.031

**Mothers' Education**	Nonliterate	16	19	1.00		1.00	
	Literate	124	41	3.59(1.69-7.63)	0.001	2.84(1.02-7.88)	0.046

COR: crude odds ratio; AOR: adjusted odds ratio; CI: confidence interval.

*∗P* value <0.05 indicates statistically significant association.

**Table 5:** Univariate and multivariate analysis of factors associated with practice of EC among female college graduating students, Harar, eastern Ethiopia, 2016 (N=200).

Variable	Category	Practice		COR (95% CI)	*P* value*∗*	AOR (95% CI)	*P* value*∗*
		Ever used	Not used				
**Age**	≤20	30	66	1.00		1.00	
	>20	36	68	2.14(1.22-3.77)	0.008	1.29(0.66-2.54)	0.449

**Marital Status**	Single	43	103	1.00		1.00	
	Ever married	23	31	2.36(1.22-4.57)	0.011	1.84(0.87-3.89)	0.113

**Knowledge**	Good	53	87	2.96(1.57-5.56)	0.001	2.53(1.29-4.92)	0.006
	Poor	13	47	1.00		1.00	

COR: crude odds ratio; AOR: adjusted odds ratio; CI: confidence interval.

*∗P* value <0.05 indicates statistically significant association.

## Data Availability

The datasets used and/or analyzed during the current study are available from the corresponding author on reasonable request.

## Abbreviations

AOR: Adjusted odds ratio
CI: Confidence interval
COR: Crude odds ratio
EC: Emergency contraception
HHSC: Harar Health Science College
IUCD: Intrauterine Contraceptive Device
RVUHC: Rift Valley University Harar Campus
SD: Standard deviation
WHO: World Health Organization.
